# Bio-Inspired Motion-Contour-Guided Visual System for Contrast-Independent Looming Perception

**DOI:** 10.3390/biomimetics11050315

**Published:** 2026-05-02

**Authors:** Junye Yao, Jinhua Zhang, Zhiyan Zhong, Huimin He, Hongxin Wang

**Affiliations:** 1 Machine Life and Intelligence Research Center, School of Mathematics and Information Science, Guangzhou University, Guangzhou 510006, China; junye@e.gzhu.edu.cn (J.Y.); hehm@gzhu.edu.cn (H.H.); 2 School of Mechanical and Electrical Engineering, Guangzhou University, Guangzhou 510006, China; 3 School of Automation, Guangdong Polytechnic Normal University, Guangzhou 510665, China; zhongzhiyan@gpnu.edu.cn

**Keywords:** bio-inspired vision, looming detection, contour evolution, contrast-independent, motion perception

## Abstract

Insects can achieve rapid and precise collision detection despite having limited neural resources. This efficiency provides a vital reference for the development of artificial collision detection systems. Existing bio-inspired models typically include LGMD-based and correlation-based methods. Methods in the former category suffer from a non-linear dependency of warning time on the object’s contrast against the background due to the strong reliance on inter-frame intensity differences. While the latter effectively describe motion perception by leveraging local motion information derived from a delay-and-correlation mechanism, they lack precise spatial boundaries, failing to isolate the actual moving target across irrelevant background dynamics. In this paper, we propose a bio-inspired visual system with a motion-contour-guided mechanism to suppress false-positive background movement while achieving contrast-independent looming warning generation. Specifically, the proposed visual system is composed of two synergistic pathways. The first pathway is designed to extract motion cues and spatial perception of motion via neuronal ensemble coding, whereas the second pathway is developed to extract the contour of the moving target by employing geometric contour evolution. By integrating this derived contour with localized motion cues, the system analyzes the dynamic evolution of the target’s boundary to identify potential collision threats. Benefiting from this fusion of structure and motion, experimental results demonstrate that the proposed visual system is more robust than conventional bio-inspired models in collision detection across distinct contrast scenarios.

## 1. Introduction

Robust collision detection serves as the foundation for the safe deployment of autonomous systems. Given the escalating toll of global traffic fatalities, the development of highly reliable collision avoidance mechanisms has become an urgent imperative. Currently, collision avoidance mainly relies on engineering solutions, such as Light Detection and Ranging (LiDAR), ultrasonic sensors, and infrared detectors. Nevertheless, these paradigms exhibit inherent limitations. Sophisticated sensors such as LiDAR are constrained by prohibitive costs and substantial power consumption, whereas simpler alternatives like ultrasonic sensors suffer from restricted detection ranges and vulnerability to environmental perturbations. Furthermore, the substantial form factor and intensive computational overhead associated with these systems severely impede their integration into resource-constrained micro-platforms. Consequently, there is a compelling demand to engineer cost-effective, energy-efficient, and miniaturized collision detection systems capable of robust performance across diverse operational platforms.

Insects serve as an exemplary biological paradigm. Despite possessing miniature brains with constrained neural resources, they have evolved highly optimized visual processing architectures. These sophisticated mechanisms enable them to isolate critical motion cues amidst dynamic background clutter and execute precise evasive maneuvers. Extensive biological reviews have demonstrated that insects can achieve robust navigation and obstacle avoidance in complex environments by utilizing limited visual inputs [1,2]. Consequently, investigating these biological visual pathways provides a fundamental basis for developing bio-inspired systems capable of robust collision detection [3].

Specifically, primary bio-inspired collision models are typically based on two specific neurons; the first is based on the Lobula Giant Movement Detector (LGMD) [4,5,6,7,8], an identified interneuron in the locust visual system that responds vigorously to looming objects [9]. Although subsequent models incorporated extra structures to enhance robustness, the core framework remains the four-layer neural network. The operational principle of these models relies on the competition between excitation and inhibition to detect potential collisions. However, a fundamental limitation of this category is that the collision warning time exhibits a non-linear dependency on the object’s contrast against the background, arising from the tight coupling of motion extraction to inter-frame intensity differences. Consequently, this vulnerability not only renders detection unreliable under fluctuating illumination but also fundamentally contradicts biological observations [10,11], which reveal that insects maintain highly consistent collision judgments regardless of varying light conditions.

Alternatively, bio-inspired collision models in the second category address motion perception through correlation-based methods, inspired by specialized visual neurons sensitive to directional motion [12,13,14,15,16]. As the classic mathematical formalization of these neural mechanisms, the Elementary Motion Detector (EMD), derived from behavioral studies of beetles and flies [12], serves as the fundamental computational unit in these models. The EMD computes local motion cues through spatiotemporal correlation of signals from adjacent photoreceptors [13,17,18,19]. These cues are then spatially integrated to compute the full-field dynamics of the visual scene. However, the primary limitation of these models is their inadequate spatial selectivity. By processing full-field dynamics rather than isolating the moving object, they are susceptible to coupling irrelevant background dynamics into the collision estimation.

Consequently, existing bio-inspired collision models face a dilemma between contrast robustness and spatial selectivity. Recent composite architectures attempt to resolve this by harmonizing motion and contrast computations in parallel pathways [20] or coupling EMDs with radial expansion filters [16]; however, their signal integration remains coupled with the magnitude of local luminance gradients, yielding temporally inconsistent warning times for kinematically identical events under varying contrasts. To the best of our knowledge, existing bio-inspired collision detection models have not adopted contour-based segmentation as a structurally independent pathway that is geometrically decoupled from pixel-level intensity, leaving this shared dependency on luminance gradients unresolved across the surveyed approaches. To resolve this, we argue that robust collision detection does not necessitate evaluating full-field dynamics. Instead, the critical motion state can be accurately determined by analyzing the temporal evolution of motion cues along the target’s contour. Biological research demonstrates that insects can perceive object contours in their visual field [11,21,22,23], and this ability is strengthened by motion signals [24,25]. Specifically, insects do not estimate the distance to the entire object, but rather to its high-contrast contour [1]. Thus, contours provide highly reliable spatial information that generalizes robustly across diverse object morphologies and motion states.

To address the limitations of conventional bio-inspired models, we propose a Motion-Contour-Guided Visual System. It consists of a motion-sensitive pathway and a contour-sensitive pathway. The first pathway extracts dense motion cues and their spatial distribution, while the second pathway focuses on capturing the target’s contour details. A subsequent collision perception module merges these two pathways to evaluate the temporal dynamics of the motion flux along the extracted contour. This effectively suppresses background noise and characterizes the precise motion state of the target to achieve robust, contrast-independent looming warning generation.

The contributions of this paper can be summarized as follows:

- We introduced a population coding mechanism involving multiple distances and directions in the motion-sensitive pathway, and optimized the direction selection for each specific pixel. This generates dense and reliable motion cues robust to localized visual interference.
- We developed a contour-sensitive pathway driven by geometric curve evolution. Coupled with the motion-sensitive pathway, it dynamically adjusts a curve to lock onto the physical boundaries of targets, thereby accurately extracting the target’s contour from complex backgrounds.
- We designed a collision perception module that integrates both pathways by calculating the motion flux along the extracted moving target contour. By evaluating the temporal dynamics of this flux, the module not only effectively reduces false alarms but also overcomes the limitation of a non-linear dependency of warning time on contrast, ensuring robust and reliable predictions across diverse motion events.

We organize the rest of this paper as follows. Section 2 briefly overviews the related work. Section 3 introduces the details of the motion-contour-guided visual system. Section 4 reports extensive experimental results together with a qualitative analysis of the outputs under different motion events.

## 2. Related Work

In this section, we briefly review related work in the areas of bio-inspired motion detection and contour perception models.

### 2.1. Bio-Inspired Motion Detection

Through long-term natural selection and evolution, animals have developed robust visual perception mechanisms to cope with collision threats. In particular, insects serve as exemplary models due to their efficient avoidance capabilities, which are governed by specialized visual neural circuits. Their performance has inspired extensive exploration and significant breakthroughs in both biological analysis and computational modeling [9,12,26]. Among these advancements, models based on the Lobula Giant Movement Detector (LGMD) have been widely deployed in robotic platforms to emulate insect-like evasive behaviors [5,27]. The core mechanism of these models relies on the critical competition between excitation and inhibition to detect potential collisions, where excitation derived from the object’s expanding edges is dynamically suppressed by lateral [5] and feed-forward [27] inhibition originating from neighboring regions activated in preceding frames [4,5,6,7,8]. However, a primary limitation of this class of models is that the collision warning time varies non-linearly with the object’s contrast against the background due to the tight coupling of motion extraction to spatiotemporal intensity changes. Consequently, without additional normalization mechanisms, this limitation significantly restricts their performance across diverse contrast environments.

Alternatively, a substantial body of research focuses on correlation-based methods inspired by Lobula Plate Tangential Cells (LPTCs) located in the lobula plate of insect visual systems [28]. Fundamentally, these methods employ delay-and-correlation algorithms to capture motion cues, generating a vector field capable of representing both direction and intensity information. For the computational implementation of this biological mechanism, researchers typically construct an LPTC sub-network, where the Elementary Motion Detector (EMD) [29] serves as the fundamental unit to process local motion cues. Functionally, a single EMD model operates by correlating signals from two adjacent photoreceptors. The signal from one channel is temporally delayed before being multiplied with the signal from its neighbor to determine the direction of movement. This mechanism allows the model to generate a strong response when an object moves in the preferred direction. Furthermore, biological studies have revealed that the insect visual system employs a parallel processing strategy, where luminance increments and decrements are handled by distinct pathways before converging at LPTC neurons [30]. Adopting this separation principle, researchers split the input luminance changes into two parallel channels, which encode luminance increments and decrements, respectively. Building on these architectures, Wang et al. [18,31,32,33,34,35] further introduced a non-linear maximization mechanism based on the collective responses of the LPTC sub-network, drawing inspiration from neurophysiology evidence [36], to prioritize strong and consistent local signals. By spatially integrating responses across the visual field and applying a winner-take-all strategy, this mechanism efficiently extracts a representative global velocity vector to infer background dynamics.

Empowered by these integrated biological mechanisms, the LPTC sub-network has also demonstrated remarkable efficacy in relevant motion detection tasks. For instance, in the domain of Small Target Motion Detectors (STMDs), which specialize in identifying the movement of small objects, Wang et al. significantly enhanced the detection capability in complex backgrounds. Their contributions include establishing direction selectivity via spatial correlations [31] and further enhancing robustness by fusing multi-cue information [32] and applying time-delay feedback loops [33].

While conventional implementations of the LPTC sub-network [18] have demonstrated remarkable success in specific tasks, they suffer from velocity ambiguity because an individual neuron’s firing rate cannot uniquely encode a specific speed. Coupled with their tendency to abstract the entire visual scene into a single, unified motion vector to infer global dynamics, they fail to provide the spatially localized motion cues required for robust collision detection. To address this limitation, we implement a pixel-wise population coding mechanism in the LPTC sub-network. By integrating the firing rates across multiple LPTCs, the system decodes precise and unique velocities. Crucially, by applying a localized maximization strategy to each pixel individually rather than pooling them globally, this coding mechanism enables the precise computation of motion direction and intensity across the entire visual field, providing the essential spatial foundation for subsequent contour evolution.

### 2.2. Contour Perception Model

Biological studies reveal that the insect visual system relies on the synergy of specific neurons for contour perception. Upstream Amacrine cells [37,38,39] first suppress the average luminance across the field of view and accentuate structural boundaries. Subsequently, the downstream Dm3-TmY circuit [25,40] decomposes the visual scene into small, orientation-specific elements, such as edges. By further integrating motion signals, this circuit effectively enhances contour recognition [11,24]. Ultimately, this coordinated neural interaction plays a critical role in their survival behaviors, such as distinguishing objects from cluttered backgrounds, avoiding collisions, and capturing prey. Because the exact neuronal circuitry underlying this synergy remains to be fully deciphered, we emulate this biological function computationally using boundary-based detection methods, guided by the coarse localization provided by the motion perception system.

To implement this synergy, we adopt active contour models [41,42,43], a mathematical framework uniquely capable of refining coarse spatial localizations into continuous collision boundaries via curve evolution. At its core, this process is formulated as an energy minimization problem, where the contour dynamically adapts under the influence of external potentials while being regularized by internal constraints. Among the various formulations of this framework, Snakes [41] and Geodesic Active Contours [44,45] are the most widely employed. Snakes are generally defined using parametric representations, which limit the solution space to predefined shapes. Because the contour evolves iteratively according to a partial differential equation, an accurate initialization is essential to ensure proper convergence [43,46]. To address these limitations, researchers developed geometric-based minimization approach known as Geodesic Active Contours. Unlike parametric models, this algorithm treats the detection process as a geometric evolution of curves, and the contour can change its structure dynamically. This non-parameterized flexibility facilitates robust contour convergence onto various object profiles without requiring strict initialization constraints.

From a mathematical perspective, the Geodesic Active Contour model aims to find a curve that minimizes a specific energy functional. This functional consists of a regularity term and a boundary attraction term, where the latter serves as an edge-stopping mechanism. The solution to this energy minimization problem is derived using the Euler–Lagrange equation [45,47], resulting in a gradient descent flow that evolves the initial contour towards the object boundaries. To solve this geometric evolution, researchers introduced the level-set formulation [48], where the curve is implicitly represented as the zero level set of a higher-dimensional function, naturally accommodating topological changes as the curve evolves. Although this formulation effectively handles topological changes by transforming the flow into a partial differential equation, a direct implementation incurs high computational costs by updating the level-set function across the entire pixel grid rather than restricting computation to the zero level set alone, which results in redundant calculations on irrelevant background pixels.

To alleviate this computational burden, various front propagation algorithms have been developed. The Narrow Band approach [49] reduces this cost by updating only pixels within a small band around the evolving front, yet it still incurs considerable overhead due to the frequent re-initialization of the band structure. Alternatively, the Fast Marching method [50] offers extreme efficiency using a heap classification algorithm to propagate the front in a single pass, but it is strictly limited to monotonically advancing fronts. To address these limitations, N. Paragios and R. Deriche [48] proposed the Hermes algorithm, which combines the flexibility of the Narrow Band with the speed of Fast Marching. This algorithm performs selective, velocity-driven local updates within a small window around the pixel with the highest propagation velocity, significantly reducing computations while preserving accuracy and supporting complex, curvature-dependent level-set propagation. Given these advantages, we employ the Hermes algorithm to robustly drive the geometric evolution in our model.

## 3. Motion-Contour-Guided Visual System

**Figure 1:**
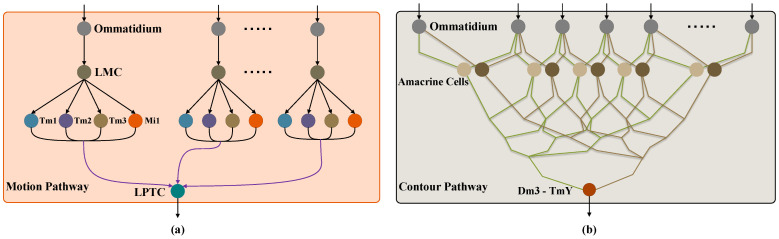
Wiring sketches of (**a**) the motion-sensitive pathway and (**b**) the contour-sensitive pathway. Colored nodes represent individual neurons. This schematic simplifies the Dm3-TmY circuit into a single block and displays only one LPTC neuron for clarity. Distinctively, while each LMC receives input from a single ommatidium, Amacrine cells integrate signals across multiple ommatidia.

As shown in Figure 1, the motion-contour-guided visual system comprises a motion-sensitive pathway and a contour-sensitive pathway. External visual stimuli are initially captured and smoothed by ommatidia in the retina layer [51]. The motion-sensitive pathway transmits the retinal output to Large Monopolar Cells (LMCs) in the lamina layer [52], followed by medulla neurons (e.g., Tm1, Tm2, Tm3, and Mi1) in the medulla layer [53], which converge onto LPTCs [26] in the lobula layer to perceive dynamic changes and extract spatial motion cues. Meanwhile, the contour-sensitive pathway initiates with Amacrine cells in the lamina layer [37,38], which receive signals derived from the ommatidia in the retina layer. Following a series of intermediate processing steps by downstream neurons, this visual information is relayed to the Dm3-TmY circuit [25,40] to extract the contour of the moving target. Finally, the collision perception module integrates the extracted structural contour with the motion cues provided by the LPTCs to evaluate the motion flux. By evaluating the temporal dynamics of this flux to determine the target’s motion state, the system ultimately generates a robust, contrast-independent looming warning. We detail the physiological functionalities and mathematical implementations of each processing stage in Section 3.1, Section 3.2 and Section 3.3.

### 3.1. Motion-Sensitive Pathway

#### 3.1.1. Retina Layer

**Figure 2:**
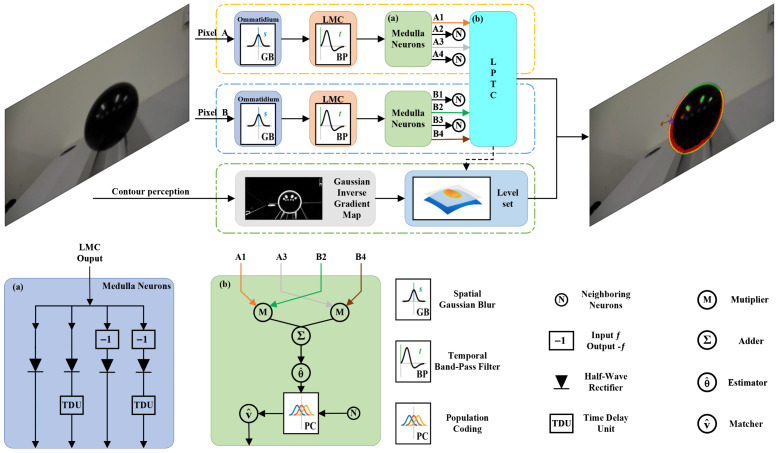
Schematic diagram of the proposed motion-contour-guided visual system (**top**), where internal structures of key components including medulla neurons (**a**) and the LPTC processing unit (**b**) are illustrated at the bottom. Each type of specialized neuron in each layer is arranged in matrix form. To simplify the presentation, only two parallel processing channels corresponding to Pixel A and Pixel B are shown. The visual signal is processed by the ommatidium and LMC layers for spatiotemporal filtering and further processed by the medulla layer. Concurrently, a contour perception channel computes Gaussian inverse gradient maps to assist contour evolution. To fuse these channels, the spatial distribution of motion is inferred by the LPTCs via population coding, which is then integrated with the level-set contour evolution. This synergistic fusion enables the system to determine the motion state of the object based on the distributed motion cues along its contour.

The retina layer is composed of many hexagonal lattices of optical units called ommatidia [23], each of which acts as an individual sampling point to receive luminance from a specific sector of the entire visual field, and sends its axons to the downstream lamina layer. A major effect of ommatidia is optical blurring, which is due to their approximate Gaussian sensitivity profile [51]. As depicted in the ommatidium module of Figure 2, we model the retina layer as a two-dimensional array of ommatidia to receive the entire image frame, using a Gaussian kernel for each ommatidium to smooth the input image. Mathematically, given an input image sequence I(x,y,t)∈R, where *x* and *y* denote the spatial coordinates and *t* represents time, the retina output of an ommatidium P(x,y,t) is formulated as the convolution of the input luminance with a Gaussian kernel:

(1) $$P\left(x,y,t\right)=\;\int \int \;I\left(u,v,t\right)\cdot G_{\sigma_{1}}\left(x-u,y-v\right)\;du\;dv.$$

The Gaussian kernel $G_{\sigma_{1}}\left(x,y\right)$ is parameterized by the standard deviation $\sigma_{1}$ and given by:

(2) $$G_{\sigma_{1}}\left(x,y\right)=\frac{1}{2\pi \sigma_{1}^{2}}\exp\left(-\frac{x^{2}+y^{2}}{2\sigma_{1}^{2}}\right).$$

Functionally, this spatial low-pass filter mimics the constrained visual resolution of insects to filter out high-frequency noise and redundant background details, yielding a smoothed and stable visual signal.

#### 3.1.2. Lamina Layer

The lamina layer is composed of large monopolar cells (LMCs), which receive inputs from the ommatidia to detect light from the same location in the visual field and then project the corresponding luminance information to the medulla layer [54]. The LMCs are highly sensitive to luminance changes over time because they hyperpolarize in response to light increments and depolarize in response to decrements [30]. Corresponding to the temporal band-pass filter shown in Figure 2, the LMC is designed to extract pixel-wise luminance variations over time, and its impulse response is characterized by the difference of two Gamma kernels. Let H(t) denote the impulse response of the LMC; then, we can write it as:

(3) $$\begin{array}{cc} H(t) & =\Gamma_{n_{1},\tau_{1}}\left(t\right)-\Gamma_{n_{2},\tau_{2}}\left(t\right), \end{array}$$

(4) $$\begin{array}{cc} \Gamma_{n,\tau }\left(t\right) & ={\left(nt\right)}^{n}\frac{\exp(-nt/\tau )}{\left(n-1\right)!\cdot \tau^{n+1}}, \end{array}$$

where $\Gamma_{n,\tau }\left(t\right)$ stands for a Gamma kernel with order *n* and time constant τ. The LMC output L(x,y,t) is defined by the convolution of the ommatidium output P(x,y,t) with H(t).

(5) L(x,y,t)=∫P(x,y,s)·H(t−s)ds.

When an object moves within the visual field, the corresponding luminance changes occur at position (x,y). This temporal fluctuation is captured by the LMC output L(x,y,t). The magnitude of this output quantifies the intensity of the change, while its polarity distinguishes the direction: positive values indicate luminance increments, and negative values signify decrements.

#### 3.1.3. Medulla Layer

The medulla layer receives the outputs from the lamina layer and processes them through two parallel channels, i.e., the ON and OFF channels, to achieve differential delays between spatial input channels and distinct processing of brightness signals [30]. Specifically, the ON channel consists of Mi1 and Tm3 neurons that selectively respond to luminance increments, with the Mi1 response temporally delayed relative to Tm3. Similarly, the OFF channel utilizes Tm1 and Tm2 neurons to encode luminance decrements, where the Tm1 response is temporally delayed relative to Tm2. As detailed in Figure 2a, based on the neural mechanisms of the medulla layer, the Tm3 and Tm2 neurons act as half-wave rectifiers that separate the LMC output L(x,y,t) based on polarity, isolating the positive and negative components, respectively. Letting $S^{\mathrm{Tm}3}\left(x,y,t\right)$ and $S^{\mathrm{Tm}2}\left(x,y,t\right)$ denote the respective outputs of these channels, we can model them as:

(6) $$\begin{array}{cc} S^{\mathrm{Tm}3}\left(x,y,t\right) & ={\left[L\left(x,y,t\right)\right]}^{+}, \end{array}$$

(7) $$\begin{array}{cc} S^{\mathrm{Tm}2}\left(x,y,t\right) & ={\left[-L\left(x,y,t\right)\right]}^{+}. \end{array}$$

The notation ${\left[x\right]}^{+}$ denotes the rectification operation max(x,0). The responses of Mi1 and Tm1 neurons, denoted as $S_{\left(n,\tau \right)}^{\mathrm{Mi}1}\left(x,y,t\right)$ and $S_{\left(n,\tau \right)}^{\mathrm{Tm}1}\left(x,y,t\right)$, serve as the temporally delayed counterparts of $S^{\mathrm{Tm}3}\left(x,y,t\right)$ and $S^{\mathrm{Tm}2}\left(x,y,t\right)$. This specific latency is mathematically implemented by convolving the source signals with a Gamma kernel $\Gamma_{n,\tau }\left(t\right)$. Formally, these delayed signals are written as:

(8) $$\begin{array}{cc} \; & S_{\left(n,\tau \right)}^{\mathrm{Mi}1}\left(x,y,t\right)=\int {\left[L\left(x,y,s\right)\right]}^{+}\cdot \Gamma_{n,\tau }\left(t-s\right)\;ds, \end{array}$$

(9) $$\begin{array}{cc} \; & S_{\left(n,\tau \right)}^{\mathrm{Tm}1}\left(x,y,t\right)=\int {\left[-L\left(x,y,s\right)\right]}^{+}\cdot \Gamma_{n,\tau }\left(t-s\right)\;ds. \end{array}$$

The time constant τ governs the delay length, while *n* specifies the order of the Gamma kernel $\Gamma_{n,\tau }\left(t\right)$.

From a computational perspective, the half-wave rectification separates the luminance increments and decrements into two independent non-negative response maps. The subsequent Gamma convolution acts as a temporal memory buffer, retaining previous motion states to facilitate the spatiotemporal correlation in the downstream layers.

#### 3.1.4. Lobula Layer

The neural outputs from the medulla layer are transmitted to T4 and T5 cells, with T4 cells receiving inputs from the ON channel neurons (Mi1 and Tm3) [54] and T5 cells receiving inputs from the OFF channel neurons (Tm1 and Tm2) [55]. Serving as the primary units for direction selectivity [56,57], these cells project their axons into four distinct layers within the lobula plate, where each layer corresponds to a specific cardinal direction of motion (front-to-back, back-to-front, upward, and downward). These direction-selective signals are then spatially integrated by the wide-field dendrites of Lobula Plate Tangential Cells (LPTCs). Consequently, LPTCs can mediate the optomotor response by integrating these local motion signals. The T4 and T5 cells correspond to the multiplier output of the Hassenstein–Reichardt model [12], which is modeled as a single Elementary Motion Detector (EMD) [29]; therefore, as structured in the LPTC sub-network in Figure 2b, we formulate the LPTC output by integrating signals from two spatially offset pixels. Let (x,y) denote the reference coordinate and $\left(x^{\prime },y^{\prime }\right)$ represent a neighboring pixel:

(10) $$\begin{array}{cc} x^{\prime }\left(\beta ,\theta \right) & =x+\beta \cdot \cos\theta ,\\ y^{\prime }\left(\beta ,\theta \right) & =y+\beta \cdot \sin\theta , \end{array}$$

where β is the distance between the two pixels and θ denotes the preferred direction of the LPTC. The output of the LPTC R(x,y,t,β,θ) is constructed by integrating the cross-correlation of luminance increments and decrements, thereby acting as a local motion detector. Specifically, the mechanism serves to multiply the non-delayed signal at the center pixel by the delayed signal at the neighboring pixel for both signal types, defined as:

(11) $$\begin{array}{cc} R(x,y,t,\beta ,\theta ) & =\underset{\mathrm{Correlation}\;\mathrm{of}\;\mathrm{Luminance}\;\mathrm{Increments}}{\underset{︸}{S^{\mathrm{Tm}3}\left(x,y,t\right)\cdot S_{\left(n_{3},\tau_{3}\right)}^{\mathrm{Mi}1}\left(x^{\prime },y^{\prime },t\right)}}\\ \; & \;+\underset{\mathrm{Correlation}\;\mathrm{of}\;\mathrm{Luminance}\;\mathrm{Decrements}}{\underset{︸}{S^{\mathrm{Tm}2}\left(x,y,t\right)\cdot S_{\left(n_{3},\tau_{3}\right)}^{\mathrm{Tm}1}\left(x^{\prime },y^{\prime },t\right)}}. \end{array}$$

where the time constant $\tau_{3}$ is set to match the time difference between the luminance signals of the two pixels, while the order $n_{3}$ determines the shape of the delayed signal.

In the context of insect optomotor responses, LPTCs exhibit a characteristic velocity optimum, where the response magnitude decreases as motion velocities increase beyond this optimum. Biological evidence suggests that velocity is encoded by a population of neurons working together, instead of by the output of a single cell [58,59]. Building on this population coding mechanism, we constructed a bank of LPTC neurons with overlapping sensitivity ranges to represent the velocity field. Specifically, the velocity selectivity of each LPTC unit is established by modulating the spatial correlation distance between the two pixels, i.e., β in Equation (11). We select a set of spatial correlation distances, denoted as ${\left\{\beta_{i}\right\}}_{i=1}^{N}$, to guarantee that motion velocities at various positions are covered by the preferred velocity ranges of the LPTCs. Subsequently, the firing rate of the *i*-th LPTC unit in response to motion at position (x,y) is defined as the maximum output along the direction $\bar{\theta }$, namely:

(12) $$\begin{array}{cc} \; & r\left(x,y,t,\beta_{i}\right)=R\left(x,y,t,\beta_{i},\bar{\theta }\left(x,y,t\right)\right), \end{array}$$

(13) $$\begin{array}{cc} \; & \bar{\theta }\left(x,y,t\right)=atan2\left(\sum_{i=1}^{N}\sin\left(\theta_{i}^{*}\right),\sum_{i=1}^{N}\cos\left(\theta_{i}^{*}\right)\right), \end{array}$$

(14) $$\begin{array}{cc} \; & \theta_{i}^{*}\left(x,y,t\right)=\underset{\theta \in \{\theta_{1},\ldots ,\theta_{n}\}}{arg max}R\left(x,y,t,\beta_{i},\theta \right). \end{array}$$

where $r(x,y,t,\beta_{i})$ denotes the firing rate of the *i*-th LPTC, $\bar{\theta }$ represents the direction of the strongest output, and $\theta_{i}^{*}\left(x,y,t\right)$ indicates the local optimal orientation that maximizes the response for the specific distance setting $\beta_{i}$. The set of firing rates ${\left\{r\left(x,y,t,\beta_{i}\right)\right\}}_{i=1}^{N}$ serves as the input for velocity estimation. By aligning these measured outputs with the LPTC tuning curves, we calculate the motion cues at position (x,y) through the following optimization equation:

(15) $$v\left(x,y,t\right)=\arg\max_{v}\prod_{i}\exp\left(-∥r\left(x,y,t,\beta_{i}\right)-f\left(v,\beta_{i}\right){∥}^{2}\right),$$

where v(x,y,t) denotes the motion cues at position (x,y) at time *t*, and $f(v,\beta_{i})$ represents the tuning curve of the *i*-th LPTC. Consequently, the components of horizontal and vertical motion cues, ϕ(t,s) and ψ(t,s), are calculated by accumulating v(t) over the time interval [s,t], defined as follows:

(16) $$\begin{array}{cc} \phi (t,s) & =\int_{s}^{t}v\left(\tau \right)\cos\bar{\theta }\;d\tau , \end{array}$$

(17) $$\begin{array}{cc} \psi (t,s) & =\int_{s}^{t}v\left(\tau \right)\sin\bar{\theta }\;d\tau . \end{array}$$

Consequently, the population coding and optimization process translates raw directional firing rates into a smooth and continuous velocity vector field, effectively mapping the precise magnitude and direction of the local dynamics.

Beyond estimating dynamic information, it is crucial to explicitly delineate the regions of perceived motion within the visual field. Specifically, LPTC responses are computed based on a set of spatial correlation distances, denoted as ${\left\{\beta_{i}\right\}}_{i=1}^{N}$, resulting in a 1×N vector of response values at each spatial location. We utilize the maximum response value, $\max(r\left(x,y,t,\beta_{i}\right))$, as the primary criterion for the spatial distribution of motion. A high value signifies a strong spatiotemporal correlation between the central and neighboring signals, reliably confirming the presence of motion. By thresholding these values, we generate a binary motion mask M(x,y,t). The coherent motion of an object, in contrast to random background noise, generates strong and contiguous responses. This process simulates the insect visual system’s capability for the coarse spatial segregation of motion. Mathematically, this thresholding operation is defined as:

(18) $$M\left(x,y,t\right)=\left\{\begin{array}{cc} 1, & \mathrm{if}\;\max_{i=1,\ldots ,N}r\left(x,y,t,\beta_{i}\right)>\delta ,\\ 0, & \mathrm{otherwise}. \end{array}\right.$$

where δ denotes the detection threshold; M(x,y,t)=1 indicates that the pixel at the location (x,y) exhibits motion characteristics, while M(x,y,t)=0 corresponds to the static background. Thus, in the spatial aggregation of pixels, M=1 provides the regions of perceived dynamics. Subsequently, both motion cues and their spatial distribution are fed into the contour-sensitive pathway for the precise extraction of the moving target’s contour.

### 3.2. Contour-Sensitive Pathway

As shown in Figure 1b, within the optic lobe of insect visual systems, the Dm3-TmY circuit serves as one of the critical structures for form vision. This circuit receives inputs from multiple neurons and comprises inhibitory Dm3 and excitatory TmY neurons that employ a lateral inhibition mechanism to generate coherent contours [25]. The ability to perceive the contour of objects is significantly enhanced by input from the motion system [11,24]. This circuit functions by decomposing the visual scene into small oriented elements to capture local structural details. Specifically, the displacement of a moving object’s single-pixel contour triggers the strongest response, which manifests as simultaneous opposite contrast polarity changes across neighboring spatial positions [23]. This suggests that the insect visual system does not perceive form as a static entity, but actively constructs it through dynamic motion cues.

To effectively distinguish moving objects from the background, our method is grounded in the biological principle that motion and form processing are not isolated but deeply intertwined [60]. Driven by this principle, our contour-sensitive pathway leverages the binary mask M(x,y,t), representing the spatial distribution of motion, as an initial spatial prior for boundary localization. However, relying solely on this motion-derived prior is insufficient for precise object contour delineation. Due to its reliance on correlating temporally delayed signals, the LPTC sub-network often yields incomplete spatial segmentation in certain situations. For example, during a diagonal looming motion (e.g., rolling down a slope from top-right to bottom-left), the object’s translation can locally counteract its geometric expansion at the boundary opposite the direction of travel. This state of optical stasis results in extremely weak luminance changes and spatiotemporal correlations at the object’s top-right corner, ultimately causing that region to be misclassified as static background.

To recover the complete contour of the moving object, we aim to emulate the capacity of the Dm3-TmY circuit in restoring contour continuity. This involves incorporating upstream signal processing mechanisms, such as Amacrine cells [37,38,39]. These cells receive signals derived from the retinal layer output of ommatidia P(x,y,t) [37] in Equation (1), and effectively attenuate the average luminance of the visual field while highlighting the structural boundaries [38,39]. In our model, we simulate this biological feature extraction using a Gaussian Inverse Gradient Map g(∇P(x,y)), which provides the essential spatial constraints for boundary refinement.

With the spatial constraints established, the subsequent contour extraction requires a reliable initialization. To achieve robust collision detection, we isolate the dominant motion region from the perceived motion map M(x,y,t), defined mathematically as the connected component containing the greatest number of motion-responsive pixels (M=1). This region represents the rough location of the moving target posing the most imminent collision threat, serving as a reliable initial position for the subsequent contour extraction of the moving target. Since the precise inhibitory–excitatory interactions within the Dm3-TmY circuit remain to be fully revealed, we abstract the computation of the Dm3-TmY circuit into a geometric optimization. Mathematically, this contour completion problem is expressed using the framework of energy minimization under the conditions of the Geodesic Active Contour problem [48]. Starting from the aforementioned initial position, an energy function is associated with the given curve and is minimized with respect to the curve’s length and position:

(19) $$E\left[C\left(p\right)\right]=\int_{0}^{1}g\left(|\nabla P\left(C\left(p\right)\right)|\right)\left|\frac{\partial C(p)}{\partial p}\right|\;dp,$$

where the first term incorporates a monotonically decreasing function g(x), which is parameterized by the standard deviation $\sigma_{2}$,

(20) $$g\left(x\right)=\frac{1}{\sqrt{2\pi }\sigma_{2}}e^{-\frac{x^{2}}{2\sigma_{2}^{2}}}.$$

The second term represents the partial derivative of the curve with respect to the parameter *p*. Conceptually, the monotonically decreasing function g(x) acts as an edge indicator, guiding the contour to anchor with structural boundaries, while the partial derivative term penalizes the curve’s arc length to enforce geometric smoothness. Driven by this energy functional, the initial curve iteratively deforms to minimize the combined integral, thereby effectively bridging the gap between the coarse motion prior and the precise structural contour.

To solve the associated Euler–Lagrange equation for this energy minimization, we utilize the level-set formulation [61]. The evolving curve *C* is implicitly represented as the zero level set of a higher-dimensional function ϕ(x,y,t)=0, which naturally handles topological changes without requiring explicit parameterization. This transforms the geometric flow into the following scalar partial differential equation:

(21) $$\phi_{t}=g(|\nabla P|)K|\nabla \phi |+\nabla g\left(|\nabla P|\right)\cdot \nabla \phi ,$$

where *t* represents the temporal progression of the evolution, driven by curvature-based smoothing and gradient-guided advection toward the boundaries; K stands for the local curvature and N signifies the inward Euclidean normal vector, both derived directly from the level-set function ϕ:

(22) $$K=\mathrm{div}\left(\frac{\nabla \phi }{\left|\nabla \phi \right|}\right),\;N=-\frac{\nabla \phi }{\left|\nabla \phi \right|}.$$

This PDE is efficiently solved using the Hermes algorithm [48,49]. Consequently, the final moving target contour $C_{final}$ is extracted as the zero level set of the evolved function ϕ.

### 3.3. Collision Perception Module

Robust collision perception requires the fusion of precise spatial boundaries and dynamic motion cues. Consequently, we integrate the outputs from both pathways. Based on the extracted target contour $C_{final}$ and the motion cues v(x,y,t) computed in the LPTC sub-network, we define the motion flux to analyze the motion state. The relationship is formulated as the line integral of the normal velocity component along the contour boundary:

(23) $$\Phi \left(t\right)=\int_{C_{final}}U\left(x,y,t\right)\cdot n\;dl,$$

where U(x,y,t) denotes the local velocity vector provided by the motion cues v(x,y,t) in Equation (15), restricted here to the extracted closed contour of the moving target $C_{final}$. n represents the unit outward normal vector along the curve $C_{final}$.

Physically, fluctuations in the magnitude of the motion flux around a stable level correspond to lateral translation. In contrast, a continuously increasing trend signifies the object’s approach or expansion. This monotonic accumulation occurs because, during a looming event, the velocity vectors are consistently oriented outward and grow in magnitude, aligning with the outward normal vectors without mutual cancellation.

To robustly predict collision risks, we evaluate the temporal dynamics of the motion flux, denoted as ΔΦ(t). Since a genuine collision event manifests as a sustained expansion, whereas transient environmental noise or momentary contour instabilities can trigger sudden but temporary perturbations in the temporal derivative, the collision alarm $A_{col}\left(t\right)$ is activated only when the temporal dynamics of the motion flux continuously exceed a critical expansion threshold $\xi_{th}$ for a persistent window of $N_{con}$ frames. This decision logic is formulated as:

(24) $$A_{col}\left(t\right)=\left\{\begin{array}{cc} \mathrm{True}, & \mathrm{if}\;\sum_{i=t-N_{con}}^{t}\Delta \Phi \left(i\right)>\xi_{th},\\ \mathrm{False}, & \mathrm{otherwise}. \end{array}\right.$$

A collision event is confirmed only when the value of $A_{col}\left(t\right)$ is evaluated as True, indicating that the looming trend has persisted for the specified duration.

## 4. Experiment

### 4.1. Experimental Setup

#### 4.1.1. Datasets

We quantify the performance of the proposed motion-contour-guided visual system on a comprehensive dataset comprising both synthetic and real-world video sequences (resolutions ranging from 480×270 to 720×480 pixels at 30 fps, with durations of 3 s to 5 s). These sequences contain diverse targets (e.g., spheres, vehicles, airplanes, and trains) and incorporate diverse motion patterns across various environments captured by a fixed camera, including lateral translation, as well as approaching and receding movements, thereby enabling systematic verification of the system’s effectiveness across distinct motion events. To assess the robustness of the collision warning generation, a subset of sequences was constructed by varying the object’s contrast against the background while preserving identical motion events. To quantify approach and recession speeds under monocular vision, targets are categorized as follows based on the duration (Δf) required for their *relative area* to change by 8%: fast-moving (Δf≤30 frames) and slow-moving (Δf>50 frames). Ground-truth collision timestamps for each sequence are manually annotated according to a consistent labeling protocol, where the collision moment is defined as the frame of physical contact between the target and the camera reference plane.

#### 4.1.2. Parameter Configuration

Parameters of the developed motion-contour-guided visual system are detailed in Table 1, which are categorized into two functional groups: the LPTC sub-network of the motion-sensitive pathway and the contour-sensitive pathway. For the LPTC sub-network, previous studies have analyzed the network’s sensitivity to core parameters [31,33,34], particularly the correlation distance β, order $n_{3}$, and time constant $\tau_{3}$. Adjusting these values changes the preferred velocity range, which consequently impacts the sensitivity of motion perception. Regarding the contour-sensitive pathway, its initialization is conditioned on the output of motion perception described above. Specifically, we dynamically set a detection threshold $\delta =0.1\times \max r(x,y,t,\beta_{i})$ to extract a coarse motion mask M(x,y,t). Based on this mask, the Hermes evolution process is initialized with a circular contour centered at the centroid of the dominant motion region, defined as the connected component containing the greatest number of motion-responsive pixels. To ensure stable convergence, the evolution is governed by a time step of Δt=0.1, a window radius of 2, and a maximum limit of 300 iterations. The remaining decision-making parameters, namely the consecutive frame count $N_{con}$ and the critical expansion threshold $\xi_{th}$, directly govern the final looming warning output. A dedicated sensitivity analysis is therefore conducted in Section 4.2 to quantitatively assess the robustness of the proposed contrast-independent looming warning generation with respect to these parameters.

#### 4.1.3. Computational Complexity and Runtime Analysis

To comprehensively evaluate the performance and real-time feasibility of the proposed visual system, we conduct a mathematical analysis of the computational complexity for processing a single image frame. The total asymptotic computational complexity of the system can be formulated as $O\left(S\cdot \left(K^{2}+D_{total}\right)\right)+O\left(I_{Hermes}\cdot L\log L\right)+O\left(P\cdot V\cdot E\right)$. This formulation is derived from the distinct functional modules comprising the proposed bio-inspired visual system.

The first stage preprocesses the input image and employs the LPTC sub-network to extract motion responses across multiple preferred spatial distances and directions. Here, *S* denotes the number of image pixels (M×N), *K* represents the spatial Gaussian kernel size, and $D_{total}$ is the sum of sampling directions across all LPTC distance settings. Because the localized spatial convolution and multi-directional cross-correlation are executed independently across the global image grid, this stage yields a linear complexity bounded by $O(S\cdot \left(K^{2}+D_{total}\right))$.

The second stage executes the Hermes algorithm to segment the moving target contour by selectively propagating the zero level set. $I_{Hermes}$ denotes the number of total propagation iterations, and *L* represents the dynamically updated number of pixels located on the active zero level set as the curve evolves. The algorithm restricts PDE evaluations to these *L* pixels and utilizes a max-heap data structure to continuously prioritize and retrieve the fastest-evolving pixels. Consequently, this scheduling yields a complexity of $O(I_{Hermes}\cdot L\log L)$.

The final stage estimates the specific velocities along the extracted target contour using the set of LPTC firing rates. *P* is the number of pixels on the target contour, *V* denotes the number of candidate velocity samples in the look-up table, and *E* represents the dimensionality of the LPTC response vector. To obtain the motion flux, the system solely calculates the Euclidean distance between the *E*-dimensional LPTC response vectors of the *P* contour points and the *V* candidates. This localized matching process results in a computational complexity of O(P·V·E) for this stage.

Preliminary runtime tests indicate that the computational bottleneck currently resides in the iterative evolution of the Hermes algorithm within the MATLAB environment, where repeated level-set propagation and active-set updates dominate the per-frame cost. The other modules introduce comparatively lower overhead. This suggests that further optimization of the Hermes evolution step could improve the overall computational efficiency of the system.

### 4.2. Parameter Sensitivity Study

The warning performance of the proposed system is governed by the two decision-making parameters in Equation (24): the consecutive frame count $N_{con}$ and the critical expansion threshold $\xi_{th}$. These parameters jointly regulate the system’s response latency and temporal stability. Two complementary metrics are employed to characterize their effects, including the *safety margin* (the temporal interval between the system-issued warning and the moment of physical collision) and the *detection consistency* across varying object contrasts.

**Figure 3:**
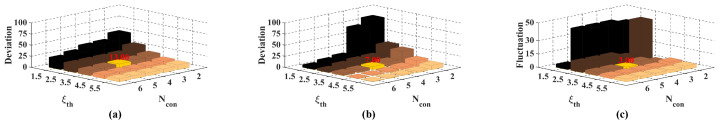
Sensitivity analysis of the decision-making parameters $N_{con}$ and $\xi_{th}$. The horizontal axes represent the consecutive frame count $N_{con}$ and the critical expansion threshold $\xi_{th}$, while the vertical axis denotes the number of frames. The bars are color-coded by row to distinguish different values of $\xi_{th}$. (**a**) The safety margin (time difference between the warning issued by the system and the actual collision time) for a slow-moving target. (**b**) The safety margin for a fast-moving target. (**c**) The detection consistency across varying object contrasts. The values marked in red indicate the performance of the selected configuration ($N_{con}=4,\xi_{th}=3.5$).

First, we fix $N_{con}=4$ to analyze the critical expansion threshold $\xi_{th}$. A comparison between $\xi_{th}=2.5$ and $\xi_{th}=4.5$ shows a clear balance between sensitivity and stability. As shown in Figure 3a, a lower setting of 2.5 gives a large safety margin of 18.7 average frames for slow motion. Similarly, Figure 3b indicates a robust safety margin of 13.0 average frames for fast motion. However, this comes at the cost of reduced detection consistency, as evidenced in Figure 3c, which shows a high fluctuation of 11 frames, meaning that a lower threshold is more susceptible to perturbations in the curve. On the other hand, increasing $\xi_{th}$ to 4.5 improves stability and reduces the fluctuation to 3 frames. However, this delays the response and leaves a risky safety margin of only 2.5 average frames for fast targets.

Next, we fix $\xi_{th}$ at 3.5 to analyze the consecutive frame count $N_{con}$. Increasing $N_{con}$ helps the system handle perturbations in the Φ(t) curve, but also adds a delay. Observing Figure 3a, as $N_{con}$ goes from 2 to 6, the safety margin clearly drops from 18.4 to 11.4 average frames for slow motion. Although different values of $N_{con}$ maintain consistent warning times across different contrasts, Figure 3b shows that a high $N_{con}$ excessively reduces the safety margin for fast targets.

Based on these analyses, we determine the optimal configuration as $N_{con}=4$ and $\xi_{th}=3.5$. This combination strikes an effective balance between sensitivity and stability. It maintains a robust safety margin of 13.0 frames for slow motion and 7.0 frames for fast motion while limiting the fluctuation to a stable 3 frames, effectively preventing false alarms.

### 4.3. Validation of Motion-Contour Mechanism

**Figure 4:**
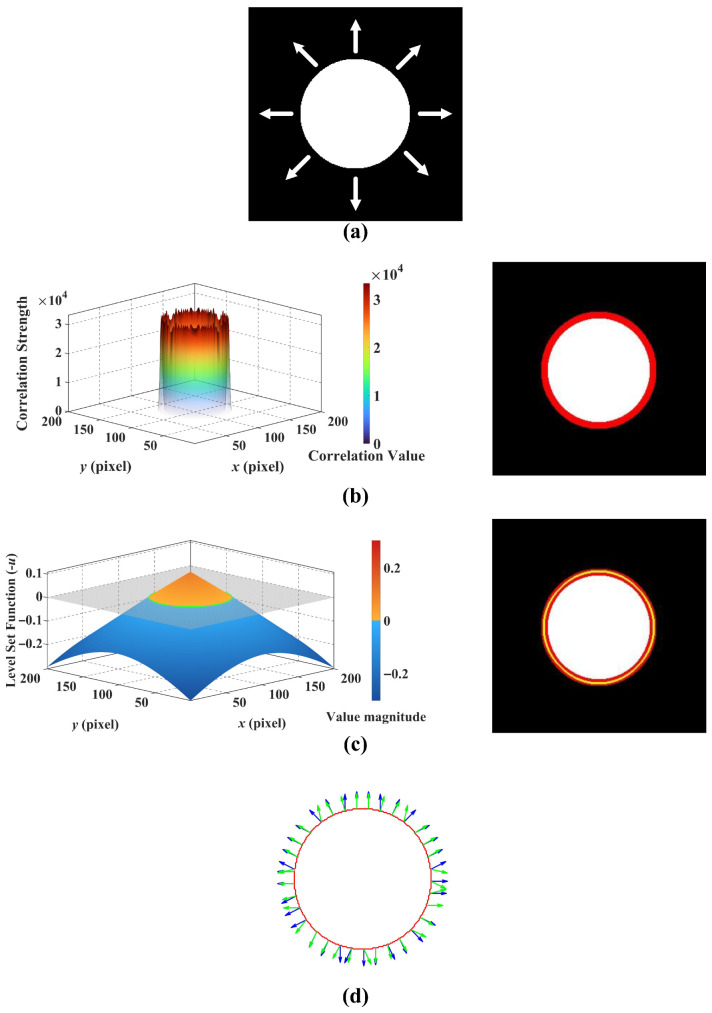
Visualization of intermediate processing results on a simulated looming sequence. (**a**) The input video sequence displays an expanding white sphere against a dark background. (**b**) Outputs of the motion-sensitive pathway: the 3D heatmap (**left**) illustrates the distribution of maximum motion response intensity, while the binary map (**right**) explicitly marks the detected motion areas in red. (**c**) Outputs of the contour-sensitive pathway: the left 3D plot visualizes the level-set function, where the intersection of the gray zero level plane with the Hermes evolution surface defines the target boundary (green curve); the right image overlays this extracted contour (yellow) onto the detected motion region. (**d**) Final motion cues along the extracted contour, where blue and green arrows denote unit normal vectors and estimated motion vectors, respectively.

To verify the efficacy of the proposed bio-inspired pathways, we conduct a layer-wise analysis of the system’s intermediate representations. Our system is inherently interpretable, with each neural layer fulfilling a distinct, physiologically grounded role. Figure 4 presents the intermediate outputs of both pathways using a simulated looming sequence, where Figure 4a illustrates the expansion of a white sphere against a dark background. In the motion-sensitive pathway (Figure 4b), the LPTC sub-network integrates spatiotemporal signals, shaping the neural response into a concentrated intensity distribution; i.e., the neural activity is maximally amplified along the moving boundary and decays rapidly toward stationary regions. Subsequently, the thresholding operation defined in Equation (18) extracts the spatial distribution of motion to form a binary mask. Building upon this motion-derived localization, the contour-sensitive pathway (Figure 4c) initializes the geometric evolution at the centroid of the dominant motion region. Under the conditions of the Geodesic Active Contour framework, the Hermes algorithm iteratively drives the level-set function to lock onto the precise contour of the moving target. Finally, Figure 4d illustrates the extracted motion cues distributed along the contour. The estimated velocity vectors are consistently oriented outward, exhibiting strong directional alignment with the unit normal vectors of the curve. Owing to this alignment, the velocity components constructively superimpose rather than undergo mutual cancellation during integration. Consequently, as formalized in Equation (23), the motion flux Φ(t) generally accumulates with the progressive expansion of the target contour, satisfying the critical expansion threshold condition that ultimately activates the looming warning.

**Figure 5:**
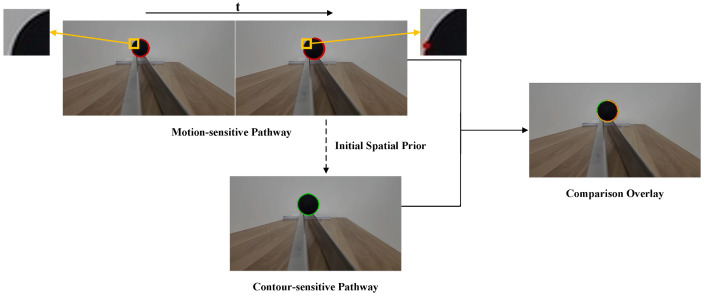
Functional validation of the proposed visual system during a diagonal looming motion (a black sphere rolling from top-left to bottom-right). In the motion-sensitive pathway, red areas represent the detected motion. The yellow boxes highlight a fixed spatial region at the object’s top-right boundary, exhibiting minimal visual change over time *t* due to translation counteracting expansion, which results in an unclosed spatial perception of motion. This detection serves as the initial spatial prior for the contour-sensitive pathway, which successfully recovers the complete physical boundary of the target (green). The Comparison Overlay illustrates the integration of both pathways, with yellow denoting the overlapping regions between the detected motion (red) and the continuous contour (green).

In contrast to the ideal symmetric expansion presented in Figure 4, where the motion-sensitive pathway successfully extracts a complete and closed boundary, real-world looming objects often exhibit asymmetric motion trajectories. Constrained by viewing angles, certain regions of the target exhibit weak luminance changes, preventing the motion-sensitive pathway from acquiring a complete and closed target boundary. Figure 5 illustrates a scenario involving a diagonal looming motion. As previously discussed, the object’s translation locally counteracts its geometric expansion at the boundary opposite to the direction of travel. This localized optical stasis yields extremely weak spatiotemporal correlations, causing the motion-sensitive pathway to fail in generating a continuous boundary, instead producing an unclosed motion contour. Relying solely on this motion-derived prior would result in fragmented spatial perception and yield invalid motion flux computations. Addressing this critical limitation, the contour-sensitive pathway leverages this unclosed detection as an initial spatial prior. Driven by the spatial constraints, the active contour algorithm bridges these structural gaps to successfully recover the complete and continuous physical boundary. Consequently, the proposed pathways are not merely parallel modules, but are tightly coupled and mutually indispensable components.

**Figure 6:**
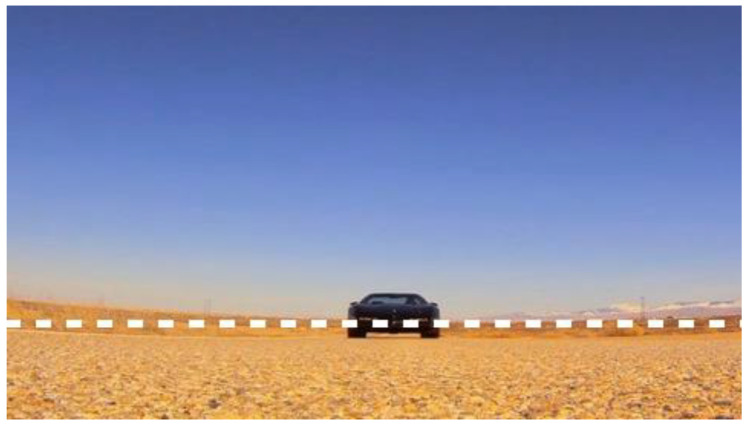
A representative frame of the input video sequence at time $t_{0}$. The scene features a fast-looming target (a black car approaching from a distance) and dynamic disturbances present in the background (e.g., wind-blown vegetation). The white dashed line indicates the fixed vertical coordinate $y_{0}=208$ selected for the signal analysis.

**Figure 7:**
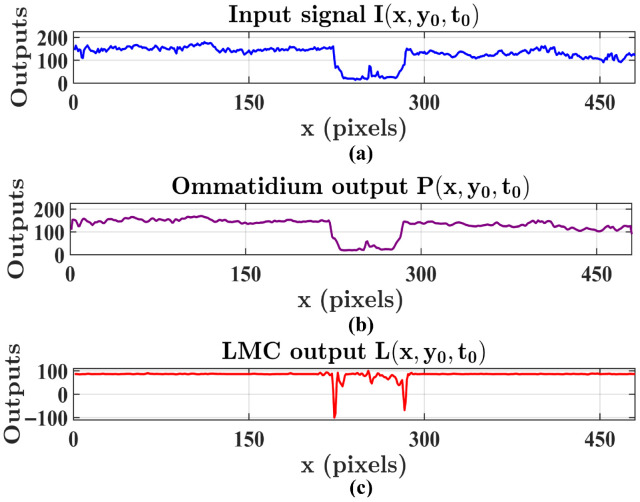
(**a**) The input signal $I(x,y_{0},t_{0})$ with respect to *x* while fixing $y_{0}=208$ pixels at a fixed time $t_{0}$. (**b**) The ommatidium output of the retina layer $P(x,y_{0},t_{0})$. (**c**) The LMC output of the lamina layer $L(x,y_{0},t_{0})$.

Having established the structural necessity of the dual-pathway architecture, we next detail the layer-wise signal processing. Consistent with the system’s inherent interpretability, we further visualize the precise signal transformations occurring at each stage of the motion-sensitive pathway. This layer-wise examination elucidates how the system effectively attenuates background noise while preserving target saliency. To intuitively visualize the signal evolution across different layers, we extract a horizontal cross-section by fixing the vertical coordinate at $y_{0}=208$ within the representative frame $t_{0}$ shown in Figure 6. Subsequently, we present neural outputs in relation to *x*, where the input signal $I(x,y_{0},t_{0})$, the ommatidium output of the retina layer $P(x,y_{0},t_{0})$, and the LMC output of the lamina layer $L(x,y_{0},t_{0})$ are displayed in Figure 7a–c. As illustrated, the input signal initially undergoes Gaussian smoothing within the ommatidium. The LMC subsequently operates as a temporal band-pass filter, extracting pixel-wise luminance variations over time. Consequently, the resulting signals exhibit distinct polarities, where positive and negative outputs encode luminance increments and decrements, respectively.

**Figure 8:**
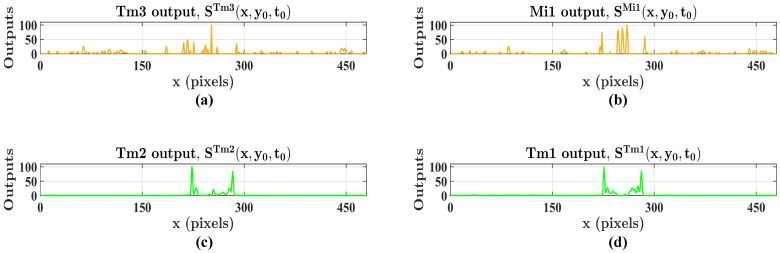
(**a**) Output of the medulla neuron Tm3, which selectively responds to luminance increases. (**b**) Output of the medulla neuron Mi1, which exhibits an integration-based latency relative to Tm3. (**c**) Output of the medulla neuron Tm2, which selectively responds to luminance decreases. (**d**) Output of the medulla neuron Tm1, which exhibits an integration-based latency relative to Tm2.

Figure 8 visualizes the signal processing within the medulla layer, where the LMC output is rectified into distinct ON and OFF channels. Figure 8a,c illustrate the instantaneous responses of Tm3 and Tm2 neurons, respectively. Because these neurons directly encode immediate luminance intensity changes in Equation (5), their spatial activation patterns are highly sensitive to transient dynamics, thereby reflecting a broader spectrum of instantaneous visual events that naturally encompass stochastic background clutter. In contrast, Figure 8b,d depict the outputs of the delayed Mi1 and Tm1 neurons. In the spatial domain, these neurons exhibit a spatially selective activation pattern, with high-intensity peaks confined to specific pixel locations corresponding to the target’s contours and internal textures, whereas responses across the remaining background regions are notably subdued. Mechanistically, this activation pattern is a direct mathematical consequence of the Gamma kernel convolution. As formulated in Equation (9), the outputs of these delayed neurons perform a continuous temporal integration, executing a temporally weighted summation of preceding luminance states. Consequently, this integration process significantly smooths out brief ambient fluctuations, effectively suppressing background clutter and robustly isolating the continuous motion features for subsequent processing.

**Figure 9:**
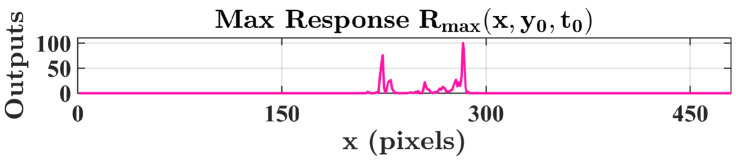
The maximum correlation output of the LPTC sub-network, denoted as $R_{max}\left(x,y_{0},t_{0}\right)$. The prominent peaks indicate the detected motion region, standing in sharp contrast to the suppressed background baseline.

These neural signals provide the foundation for the subsequent motion perception stage. Based on the correlation defined in Equation (11), the movement of the target is captured by correlating the instantaneous response at a pixel (x,y) with the delayed response from a neighboring pixel $\left(x^{\prime },y^{\prime }\right)$ located at a distance β along the direction θ. This correlation process significantly amplifies the response within the motion region, yielding the maximum response ($R_{max}$) profile visualized in Figure 9. Meanwhile, the background regions maintain a near-zero baseline due to the suppression of noise in the input streams. Through this cascade of neural processing stages, the proposed system reliably extracts spatially consistent motion cues from cluttered environments, providing a reliable initialization prior for subsequent contour evolution.

**Figure 10:**
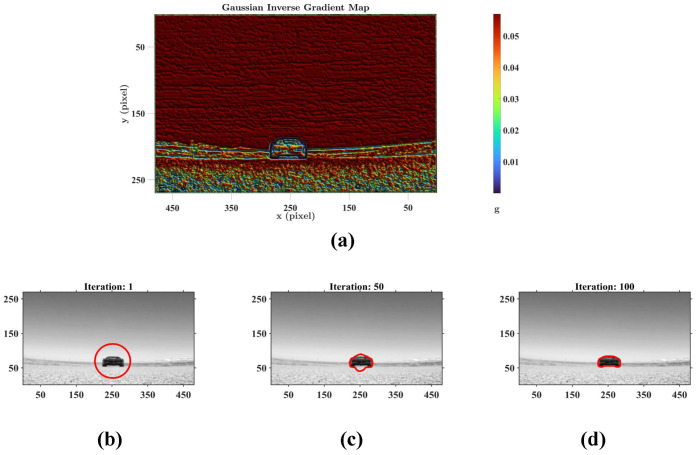
Visualization of the contour evolution process. (**a**) The Gaussian inverse gradient map, where low values (blue regions) correspond to object boundaries. (**b**–**d**) The iterative evolution of the level-set function.

**Figure 11:**
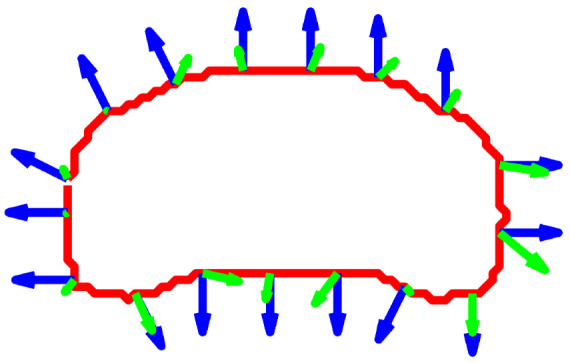
Visualization of extracted motion cues along the contour: The blue arrows represent the unit normal vectors, while the green arrows denote the estimated motion vectors. The velocity vectors on the left side appear relatively weaker in magnitude due to the specific viewing angle of the camera, which results in a perspective projection effect.

Having established the localization of the motion region by the motion-sensitive pathway, we now focus on the contour-sensitive pathway, which refines this coarse motion perception into a precise object contour. To validate the mechanism of geometric evolution, Figure 10 visualizes the intermediate states of the level-set function driven by the Hermes algorithm. Consistent with the physiological role of Amacrine cells in suppressing average luminance while accentuating structural boundaries [39], the Gaussian Inverse Gradient Map in Figure 10a is constructed to implement an analogous function. This map exhibits low values at the target contour and remains high in homogeneous regions. Consequently, it serves as the edge-stopping term within the Geodesic Active Contour formulation, causing the evolving curve to decelerate and arrest at the target contour with high precision. Figure 10b–d illustrate the iterative evolution process, which is initialized at the centroid of the dominant motion region detected by the motion-sensitive pathway. The zero level set progressively converges upon the physical boundary of the target vehicle, demonstrating robust convergence behavior under the Hermes-driven geometric evolution. This outcome is consistent with the functional role of the Dm3-TmY circuit, where spatial information from motion is instrumental in refining contour perception, analogous to how our motion mask guides boundary convergence. Collectively, these results substantiate the system’s capacity to extract the precise geometric features required for the subsequent motion flux computation, as further corroborated by Figure 11. The estimated velocity vectors demonstrate strong directional alignment with the outward unit normal vectors along the contour, confirming that the system accurately captures the object’s looming expansion.

Building on this layer-wise interpretability analysis, the subsequent sections present quantitative evaluations of the system’s motion selectivity and contrast-independent warning performance across diverse real-world scenarios.

### 4.4. Evaluation of Motion Selectivity and Contrast-Independent Performance

To further validate the proposed motion-contour-guided visual system under real-world conditions, experiments are conducted on recorded video sequences comprising two primary scenarios: controlled indoor ball-rolling tests and outdoor vehicle approach sequences. Compared with the controlled indoor settings, real physical environments introduce significant background noise, such as glare, shadows, and complex textures. Furthermore, objects in outdoor scenes exhibit unconstrained and complex dynamics, in contrast to controlled motion profiles observed in indoor tests. This unpredictability constitutes a realistic stress test for the system’s detection stability and temporal consistency.

**Figure 12:**
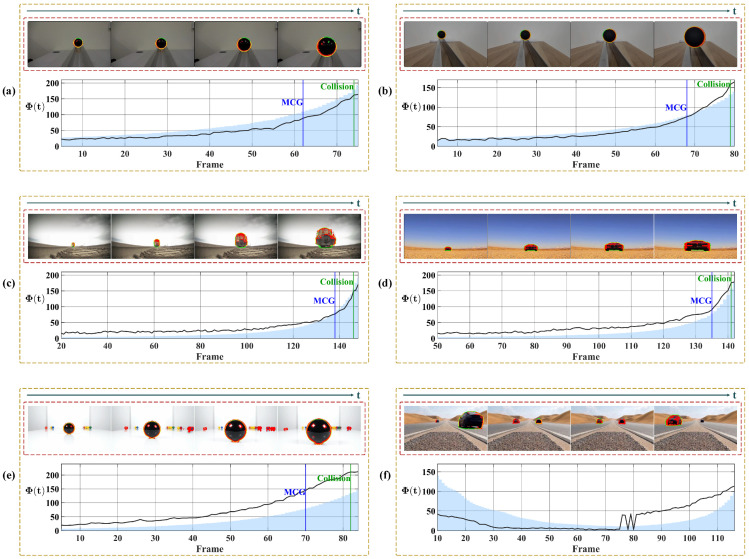
System responses to approaching objects against diverse real-world backgrounds. In each subfigure, the light blue histogram displays the temporal variation of the object’s size. The solid black curve tracks the motion flux along the contour Φ(t). Vertical markers indicate critical timing events: the blue line represents the looming warning time generated by the proposed system, while the green line indicates the exact occurrence of the collision. Consequently, an effective collision warning is inherently required to occur ahead of this temporal boundary. Color overlays denote perceived motion (red), extracted contours (green), and their overlap (yellow). (**a**) A black sphere approaching at a moderate speed toward the bottom left. (**b**) A black sphere approaching at a moderate speed toward the bottom right. (**c**) A train approaching at high speed. (**d**) A black vehicle approaching at high speed. (**e**) A black sphere approaching amidst multiple moving distractors. (**f**) A receding vehicle followed by an approaching one on a lateral trajectory.

Figure 12 shows the quantitative results of the looming detection experiments. In the early detection phase, when the target remains at a large distance, the motion flux response fluctuates at a low baseline rather than remaining identically zero. To ensure detection stability, the warning mechanism is activated only when the temporal derivative of motion flux ΔΦ(t) exceeds the prescribed threshold over several consecutive frames, thereby suppressing transient perturbations and eliminating false alarms. As the target progressively approaches, the system operates robustly across a range of approach velocities. Under the controlled motion profiles of the indoor scenarios (Figure 12a,b), Φ(t) exhibits a sustained ascending trend, successfully anticipating the collision event by triggering a warning approximately 10 frames in advance. In contrast to the controlled indoor scenarios, the unconstrained outdoor vehicle scenarios (Figure 12c,d) involve significantly higher approach velocities, causing the object’s projected area to undergo rapid expansion. Despite this abrupt looming onset, Φ(t) rises sharply and triggers a warning approximately 5 frames before impact. This reduced frame margin objectively illustrates the faster looming progression compared to the controlled indoor settings. Building upon these single-target scenarios, we further evaluated the system’s performance in looming experiments involving multiple simultaneously moving objects to verify its selective attention. As shown in Figure 12e, in the presence of multiple distractors moving irregularly, the looming target consistently maintains its status as the dominant motion region due to its progressive expansion, allowing the model to remain locked on the primary threat and trigger a timely warning 11 frames before the potential collision. Conversely, in Figure 12f, the system demonstrates an adaptive tracking capability by shifting the potential threat focus from a receding vehicle to a looming one as the dominant motion region changes. Since the latter poses no direct head-on threat, the temporal dynamics of the motion flux correctly remain below the critical expansion threshold. Collectively, these results confirm that the system not only maintains reliable collision perception across varying approach velocities, but also effectively mitigates multi-object interference by isolating the dominant motion region.

**Figure 13:**
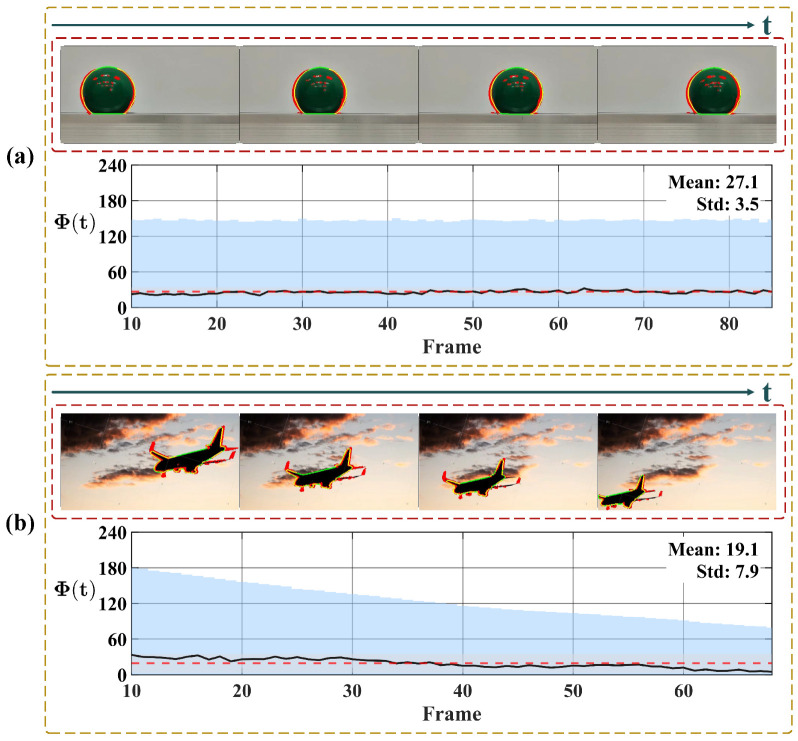
System responses to translational motion events. The light blue histograms and solid black curves represent the object size and the computed motion flux Φ(t), respectively. The mean and standard deviation (Std) displayed in the top-right corner quantify the stability of Φ(t). (**a**) Lateral translation of a green sphere. (**b**) An airplane executing a compound maneuver of translation and receding motion.

To further verify the system’s motion selectivity, Figure 13 illustrates the response to translational motion. Unlike the progressively increasing trend characteristic of looming events, Φ(t) here maintains a low baseline throughout. In the pure translation scenario (Figure 13a), the curve fluctuates horizontally around a stable baseline, yielding statistical results with a low mean and small standard deviation, confirming the system’s effective suppression of non-threatening lateral motion. In contrast, the second scenario (Figure 13b) introduces a receding motion component alongside translation. Consequently, the curve exhibits a distinct downward trend in addition to the fluctuations. Furthermore, when portions of the target exit the image frame, the computable motion contour is reduced, resulting in a corresponding decrease in the integrated response Φ(t). This behavior is physically expected, as it reflects the loss of valid motion cues along the contour rather than a system artifact.

**Figure 14:**
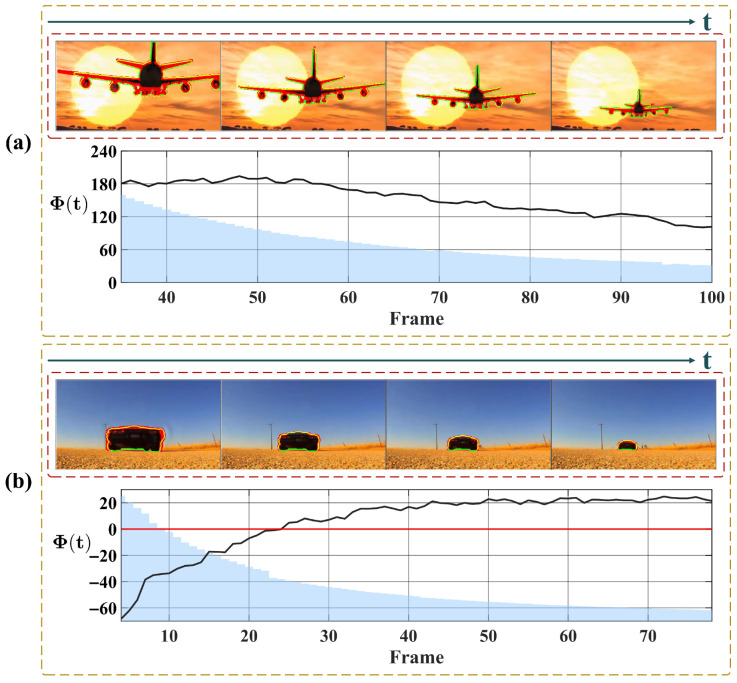
System responses to receding motion events. The light blue histograms represent the object size, and the solid black curves denote the computed motion flux Φ(t). (**a**) An airplane receding at a slow speed; (**b**) A vehicle receding at a high speed.

Subsequently, we analyze the system’s response to receding motion in Figure 14. The results reveal that the detection of retreating objects is non-uniform and depends on the specific motion composition. Figure 14a presents a slowly receding object. Although the curve exhibits a downward trend, the values remain positive throughout. This occurs because the motion is dominated by lateral translation rather than radial contraction; consequently, the velocity vectors along the boundary largely maintain an outward orientation, accumulating a positive integral. In contrast, Figure 14b depicts a rapidly receding target. In the initial phase, the curve exhibits a pronounced negative excursion. This distinct negative response arises because the rapid visual contraction generates inward-pointing motion vectors, which directly oppose the outward unit normal vectors. However, as the sequence progresses, the curves in both scenarios tend to stabilize. This stabilization occurs as the targets recede into the distance, their projected areas on the image plane shrink drastically, and the magnitude of the visual contraction diminishes. At this stage, the integral Φ(t) is no longer driven by strong directional motion cues but is sustained by residual background noise and minor boundary fluctuations, resulting in steady oscillation.

**Figure 15:**
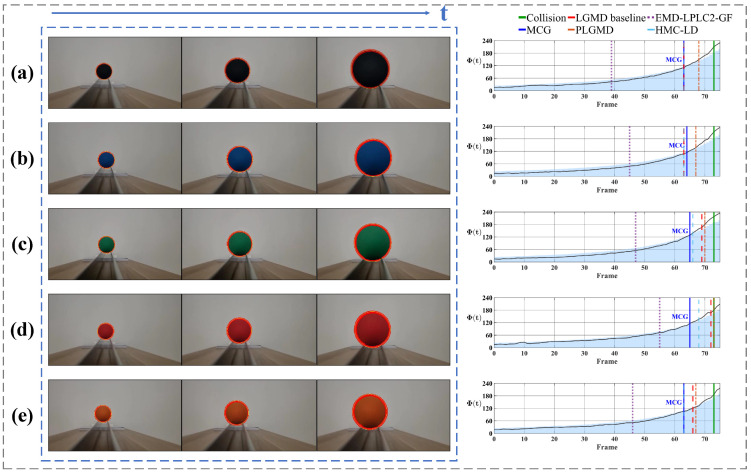
System responses to the same approaching event (sphere) under varying contrast conditions. In each subfigure, the light blue histogram displays the temporal variation of the object size. The solid black curve tracks the motion flux along the contour Φ(t). Vertical markers indicate critical timing events: the blue solid line for the proposed system (MCG), red dashed line for the LGMD baseline model, orange dash-dot line for PLGMD, purple dotted line for EMD-LPLC-GF-model, and cyan fine-dashed line for HMC-LD. The green line marks the moment of physical collision. To ensure successful evasion, the effective warning time issued by our system must strictly precede this green marker. Subfigures (**a**–**e**) correspond to the same approaching object with different Weber contrasts.

**Figure 16:**
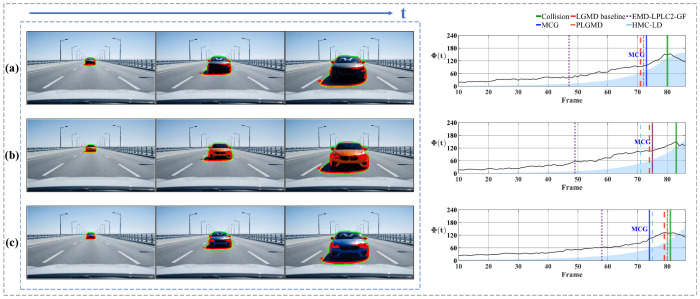
System responses to synthetic collision events involving approaching vehicles of different colors on a cross-sea bridge. In each subfigure, the light blue histogram displays the temporal variation of the object size. The solid black curve tracks the motion flux along the contour Φ(t). Vertical markers indicate critical timing events: Blue solid line for the proposed system (MCG), red dashed line for the LGMD baseline model, orange dash-dot line for PLGMD, purple dotted line for the EMD-LPLC-GF-model, and cyan fine-dashed line for HMC-LD. The green line marks the moment of physical collision. As required for collision avoidance, the proposed system must trigger its warning time strictly prior to this green marker. Subfigures (**a**–**c**) correspond to similar approaching vehicles with different colors.

Finally, we evaluated the contrast-independent performance of the proposed system. Figure 15 and Figure 16 present the system responses to approaching events under varying contrast conditions. The former depicts an indoor ball-rolling experiment, while the latter shows a synthetic vehicle approaching on a direct collision course. Across both scenarios, the critical warning timing events demonstrate high consistency. As illustrated in Figure 15, the proposed system achieves an average looming warning time of approximately 9 frames prior to the actual collision, exhibiting only minor fluctuations across multiple trials. Similarly, for the sequences depicted in Figure 16, the proposed system achieves an average warning time of 6.7 frames prior to impact, with the timing fluctuating by 2 frames. Such robustness is attributed to the system’s high reliance on the moving target’s contour, rendering its performance invariant to variations in object contrast.

**Figure 17:**
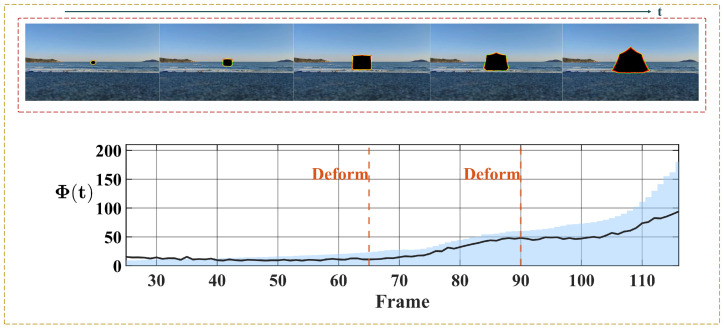
Failure case of a deforming looming target failing to trigger a warning. The top sequence shows a simulated object expanding against a fixed background, deforming from a circle to a square (frame 65) and then to a polygon (frame 90). In the bottom plot, the light blue histogram and solid black curve represent the temporal variation of the object size and the contour-based motion flux Φ(t), respectively, with orange dashed lines indicating deformation onset.

To provide a comprehensive and balanced evaluation of the proposed method, we finally present a failure case analysis to discuss the limitations of the system under unconstrained conditions. As illustrated in Figure 17, we simulate a scenario where a looming target undergoes structural deformations against a fixed real-world background. During these deformation phases, the expansion of the object is significantly disrupted, and the increase in its size becomes marginal. Since our system relies on the integration of motion cues along the valid contour, the inward-pointing or lateral velocity components induced by this local structural contraction cancel out the genuine outward-pointing expansion vectors along the contour. Consequently, even though the target is macroscopically approaching, the increase in the motion flux Φ(t) remains limited. As a result, the temporal dynamics of the motion flux fail to continuously exceed the critical expansion threshold $\xi_{th}$, which ultimately prevents the system from successfully triggering a looming warning. This analysis reveals a specific boundary condition of the current visual system, indicating its potential vulnerability when dealing with targets exhibiting non-rigid structural deformations during their approach.

### 4.5. Quantitative Comparison with Biomimetic Models

Having established the robustness and contrast-independence of the proposed system under various scenarios, we now present a quantitative benchmark against several recent representative biomimetic visual models: the classic LGMD baseline [4,5,6], the probabilistic noise-filtering PLGMD [8], the correlation-based EMD-LPLC2-GF [16], and the contrast-normalized HMC-LD [20]. To provide a holistic evaluation, the quantitative results presented in Table 2 assess the models across four interconnected dimensions. The model’s spatial–temporal sensitivity is jointly quantified by the *Area at Warning* (the proportion of the field of view occupied by the target at the exact moment the collision warning is triggered) and the anticipatory *Warning Lead Time* prior to physical impact. Furthermore, we evaluate the system’s detection consistency via the *Coefficient of Variation* (the ratio of the standard deviation to the mean of warning times across targets with identical motion events but varying contrasts), alongside the overarching *Error Rate* that encompasses both missed collisions and false alarms.

As detailed in the quantitative results, the proposed system achieves balanced and robust performance across all four evaluation dimensions. Specifically, it triggers collision warnings when the approaching target occupies approximately 7.988% of the visual field, ensuring an average safety margin of 8.667 frames prior to actual impact, while maintaining a competitive error rate of 12.834%. Most notably, the proposed system records the lowest Coefficient of Variation at 1.747%, demonstrating superior temporal consistency across all evaluated contrast conditions.

This temporal stability stands in marked contrast to the warning time deviations exhibited by other bio-inspired models under varying contrast levels. This performance discrepancy fundamentally arises from a shared structural limitation in their underlying detection mechanisms. Whether relying on inter-frame pixel intensity differences (as in the LGMD baseline and PLGMD) or contrast-modulated local motion computations (as in the EMD-LPLC2-GF), the strength of their internal response is intrinsically coupled with the target’s visual contrast. Consequently, a highly contrasting object generates a stronger response and crosses the warning threshold prematurely, whereas a low-contrast object produces a weaker response and delays the warning, leading to inconsistent warning timing. The EMD-LPLC2-GF exemplifies this sensitivity. While its correlation-based architecture allows for an exceptionally early average warning lead time of 30.583 frames, this hypersensitivity to local luminance changes results in the highest temporal fluctuation, with a CV of 10.180%. Recent models have attempted to mitigate this issue. For instance, PLGMD introduces probabilistic network connections to smooth out response fluctuations, and HMC-LD employs dynamic contrast-inhibition mechanisms to neutralize excessive luminance excitations. While these methods successfully reduce their Coefficient of Variation to 3.809% and 3.318%, respectively, compared to the LGMD baseline’s 4.836%, they still derive their foundational motion cues from pixel-level intensity and therefore cannot completely eliminate the timing shifts induced by contrast variations. Conversely, the proposed system evaluates collision risks by tracking the physical contour of the target, thereby significantly reducing its reliance on luminance variations. From a geometric perspective, the contour of an approaching object expands outward at a consistent rate determined solely by its speed, regardless of whether the object is dark or light. By exploiting this pure geometric expansion, the proposed system ensures contrast-independent looming warning generation.

## 5. Conclusions and Discussion

In this paper, we propose a motion-contour-guided visual system for robust collision detection in varied environments. The key contributions of the proposed system are threefold. First, a population-coded motion-sensitive pathway is introduced to generate dense and reliable motion cues, replacing the full-image spatiotemporal intensity differences relied upon by traditional models. Second, a geometric-curve-driven contour-sensitive pathway is developed to extract the physical target boundary, providing a structurally grounded representation that is decoupled from pixel-level contrast. Third, by computing the motion flux as a line integral of normal velocity components along the extracted contour, the system quantifies target expansion in a contrast-independent manner, effectively suppressing background noise, reducing false alarms, and overcoming the non-linear dependency of warning time on contrast. Experimental results demonstrate that the proposed system achieves consistent and contrast-independent collision prediction across diverse real-world scenarios.

Despite these promising capabilities, the current framework serves as a proof-of-concept with specific computational and operational constraints. Computationally, while the theoretical complexity is bounded, the system is currently evaluated in a MATLAB 2024a environment where the iterative level-set evolution of the contour-sensitive pathway constitutes an execution bottleneck, precluding deployment on resource-constrained embedded platforms. Operationally, the system is restricted to stationary-camera scenarios. Under this setting, the LPTC sub-network responds exclusively to independently moving objects; any camera ego-motion would generate widespread global motion responses that, without an intrinsic decoupling mechanism, would inevitably mask the target’s local expansion and hinder accurate contour extraction. Additionally, as revealed by the failure case analysis, the system’s reliance on contour integration renders it vulnerable to targets exhibiting non-rigid deformations, where localized structural contractions may offset looming expansion cues.

In terms of practical applicability, the proposed system in its current form is directly suited to stationary-camera deployment contexts. Representative scenarios include fixed infrastructure-mounted collision warning systems, ground-based surveillance platforms requiring contrast-robust target detection, and bio-inspired robotic systems operating in controlled or semi-structured environments. Each of these settings benefits from the system’s core capability of contrast-independent looming detection without reliance on global image intensity differences.

These limitations motivate several directions for future investigation, each grounded in a concrete deployment target. To enable integration into micro-aerial vehicles and autonomous ground robots, future work will explore algorithmic optimization of the Hermes contour evolution and model acceleration strategies targeting the identified computational bottleneck. To extend applicability to vehicle-mounted collision avoidance and drone navigation systems, ego-motion compensation and global background subtraction strategies will be investigated. Furthermore, to accommodate articulated or flexible targets in unstructured real-world environments, additional biologically motivated mechanisms will be explored, including attention-based modulation, deformation-aware adaptive flux integration, and border ownership coding, with the aim of continuously enhancing the robustness and accuracy of the visual system under highly dynamic and challenging conditions.

## Figures and Tables

**Table 1:** Parameters of the motion-contour-guided visual system.

Equation	Parameters
(1)	$\sigma_{1}=1$
(3)	$n_{1}=2,\tau_{1}=3,n_{2}=6,\tau_{2}=9$
(10)	β∈{2,4,6,8,10,12,14,16,18}
(11)	$n_{3}=25,\tau_{3}=30$
(20)	$\sigma_{2}=7$
(24)	$N_{con}=4,\xi_{th}=3.5$

**Table 2:** Quantitative comparison of the proposed system against representative biomimetic models across four performance dimensions. The Coefficient of Variation is computed as the ratio of the standard deviation to the mean of warning times across targets with identical motion events but varying contrasts. Error Rate encompasses both missed detections and false alarms.

Model	Area at Warning	Warning Lead Time (Frames)	Coefficient of Variation	Error Rate
LGMD baseline	8.115%	9.833	4.836%	16.320%
PLGMD	9.018%	7.583	3.809%	13.518%
EMD-LPLC2-GF	3.531%	30.583	10.180%	14.129%
HMC-LD	7.232%	11.545	3.318%	12.452%
MCG (Ours)	7.988%	8.667	1.747%	12.834%

## Data Availability

The data used to support the findings of this study are included within the article.
